# Machine Independence of Ultrasound-Based Quantification of Vitreous Echodensities

**DOI:** 10.1167/tvst.12.9.21

**Published:** 2023-09-26

**Authors:** Cameron Hoerig, Justin H. Nguyen, Jonathan Mamou, Cedric Venuat, J. Sebag, Jeffrey A. Ketterling

**Affiliations:** 1 Weill Cornell Medicine, Department of Radiology, New York, NY, USA; 2 VMR Institute for Vitreous Macula Retina, Huntington Beach, CA, USA; 3 Quantel Medical, Cournon d'Auvergne, France; 4 Doheny Eye Institute/Geffen School of Medicine/UCLA, Los Angeles, CA, USA

**Keywords:** vitreous, ultrasonography, quantitative ultrasound (QUS), vision degrading myodesopsia (VDM), floaters

## Abstract

**Purpose:**

Quantitative ultrasound (QUS) provides objective indices of Vision Degrading Myodesopsia (VDM) that correlate with contrast sensitivity (CS). To date, QUS methods were only tested on a single ultrasound machine. Here, we evaluate whether QUS measurements are machine independent.

**Methods:**

In this cross-sectional study, 47 eyes (24 subjects; age = 53.2 ± 14.4 years) were evaluated with Freiburg acuity contrast testing (%Weber), and ultrasonography using 2 machines: one with a 15-MHz single-element transducer and one with a 5-ring, 20-MHz annular-array. Images were acquired from each system in sequential scans. Artifact-free, log-compressed envelope data were processed to yield three parameters (mean amplitude, *M*; energy, *E*; and percentage filled by echodensities, *P50*) and a composite score (*C*). A B-mode normalization method was applied to the 20-MHz datasets to match QUS parameters at both frequencies. Statistical analyses were performed to evaluate correlations among CS, *E*, *M*, *P50*, and *C* for both machines.

**Results:**

QUS parameters from each machine correlated with CS (*R* ≥ 0.57, *P* < 0.001) and there was correlation between machines (*R* ≥ 0.84, *P* < 0.001). Correlations between CS and QUS parameters were statistically similar for both machines (*P* ≥ 0.14) except when the 20-MHz data were normalized (*P* = 0.04). Reproducibility of QUS parameters computed from 20-MHz data were satisfactory (52.3%–96.3%) with intraclass correlation values exceeding 0.80 (*P* < 0.001).

**Conclusions:**

The high correlation between QUS parameters from both machines combined with a statistically similar correlation to CS suggests QUS is an effective, machine-independent, quantitative measure of vitreous echodensities.

**Translational Relevance:**

QUS may be applied across clinical ophthalmic ultrasound scanners and imaging frequencies to effectively evaluate VDM.

## Introduction

The vitreous body is a hydrated extracellular matrix that forms an optically and acoustically transparent gel filling the posterior segment of the eye.^1^ With myopia and age, liquefaction causes an increase in liquid vitreous and decrease in the volume of the gel-like matrix. Collagen-hyaluronan interaction during the liquefaction process can produce collagen fibril aggregates to form within the vitreous body that scatter light and cause the clinical phenomenon of floaters.^2^^–^^4^ Pathologies, such as diabetes and myopia,^5^^,^^6^ can increase the rate of vitreous liquefaction and the formation of intravitreal collagen aggregates. Age-related posterior vitreous detachment (PVD) causes acute floaters due to light scattering by the dense collagen matrix of the posterior vitreous cortex and its irregular surface interfering with light transmission to the retina.

Vitreous opacification is known to degrade the contrast sensitivity (CS) and quality of life (QOL).^6^^–^^8^ Although vitreous floaters do not constitute a clinically significant condition for all patients, many experience a considerable reduction in QOL that necessitates clinical intervention. Vitreous opacification that degrades CS is referred to as vision degrading myodesopsia (VDM). Interventions for VDM include vitrectomy, neodymium:yttrium-aluminum-garnet (YAG) laser vitreolysis, or (potentially) pharmacologic vitreolysis. Until about 1 decade ago, evaluating the quantity of vitreous opacification and the impact on vision and QOL has largely relied on subjective evaluations and the decision to pursue treatment has historically not been based on quantitative measures. Moreover, there was a lack of quantitative outcome measures to evaluate treatment success.

Abnormal vitreous not only disrupts the transmission of photons to the retina, but also forms echodensities that provide acoustic contrast. The relationship between light scattering and acoustic contrast has recently been identified.^9^ Our past studies developed quantitative ultrasound (QUS) methods to provide objective measures of vitreous echodensity that correlate with CS and QOL, the latter measured by the National Eye Institute Visual Function Questionnaire (NEI VFQ).^8^^,^^10^^–^^12^ We established that QUS methods provided effective quantitative parameters to assess pre- and post-treatment states of vitreous.^10^ CS and QUS metrics have been used to evaluate the effects of YAG Laser Vitreolysis^11^ and vitrectomy^7^ in patients with VDM. These studies point to the need to evaluate treatment outcomes in a quantitative fashion so that better treatments can be developed to cure VDM.

The goal of QUS is to generate quantitative output parameters of tissue microstructure that are machine- and operator-independent.^13^ QUS methods using raw radiofrequency (RF) echo data, such as those based on normalized measurements of the backscatter coefficient, typically have a rigorous mathematical foundation^13^^–^^15^ and have successfully been applied to assess acoustic properties of different organs and tissue types.^13^^,^^16^^,^^17^ Although we have demonstrated that our QUS approach provides a useful objective assessment of vitreous echodensities, we were constrained to utilize log-compressed envelope data which precludes standard normalization approaches based on RF data. Our prior studies were also conducted using a single machine and probe combination and those methods have not been tested on a different clinical ultrasound scanner.

The goal of this study is to determine whether our QUS vitreous echodensity parameters are consistent in different ophthalmic ultrasound machines with different probe geometry and center frequency. The original studies were conducted with a single-element, 15-MHz transducer. The present study uses an advanced, 5-element annular-array transducer having a higher center frequency of 20 MHz. A cohort of subjects with varying levels of CS and VDM were sequentially scanned using both ultrasound probes and machines. We statistically analyzed the results to determine how well the QUS parameters correlated between the two machines, factoring in the variability that can occur from repeated measurements with the same system in a single session. Furthermore, we performed a perturbation analysis to determine the number of images necessary to achieve stable estimates of QUS parameters with low variance, a necessary step to guide future applications of QUS to assess vitreous echodensities.

## Methods

This cross-sectional study was approved by the Institutional Review Board of St. Joseph Hospital, Orange, California. All research adhered to the tenets of the Declaration of Helsinki and the Health Insurance Portability and Accountability Act. Informed consent was obtained from each participant.

### Subjects

There were 47 eyes in 24 patients (16 men and 8 women; mean age = 53.2 ± 14.1 years) with varying levels of VDM enrolled in this study. No patients had a medical history of vitreo-retinal surgery or YAG laser treatment, and pseudophakic subjects had cataract surgery a minimum of 12 months prior to study entry.

### Visual Function

Visual function was evaluated by measuring the CS of each eye using computer-based Freiburg Acuity Contrast Testing (FrACT),^18^^–^^20^ as previously described.^7^^,^^21^ FrACT uses a light-emitting diode display monitor to display a “tumbling” monochromatic Landolt C optotype. All subjects were tested at a distance of 2.9 m in a dark room after 3 minutes of dark adaptation. CS values from the test are reported in terms of the Weber index:

(1) $$\begin{array}{c} \%W=\frac{Luminance_{max}-Luminance_{min}}{Luminance_{max\;}}\times \;100\%,\; \end{array}$$

where larger values of %*W* represent worse CS. This metric has been found to be repeatable^7^^,^^21^ and consistent with VFQ,^10^ making it a useful measurement for quantifying visual function.

### Ultrasonography

Two clinically approved ophthalmic ultrasound machines (Quantel Medical, Cournon d'Auvergne, France) were used at the VMR Institute for Vitreous Macula Retina (Huntington Beach, CA, USA). Measurements of the acoustic field of both transducers demonstrated derated spatial-peak, temporal average intensity, and mechanical index within the US Food and Drug Administration limits for ophthalmic ultrasound machines. The first machine (Aviso) was used in previous studies performing QUS to assess vitreous echodensities.^7^^,^^10^^,^^11^ It used a 15-MHz single-element, focused probe with a 6-dB bandwidth of 9.5 to 20 MHz. The transducer had a 23-mm focal length and a 7-mm diameter. Customizations to the system permitted exporting log-compressed echo envelope data sampled at 40 MHz for later QUS processing. The second machine (Absolu) utilized a 20-MHz annular-array transducer comprising 5 individually excitable rings. The geometric focal length was 22 mm and the full aperture diameter was 9 mm. Echo data were collected by transmitting and receiving on all five rings simultaneously. In this mode, the -6-dB bandwidth spanned 11.1 to 24.6 MHz. Log-compressed echo envelope data were sampled at 60 MHz and exported for QUS processing. All echo data exported from the Aviso or Absolu machine had identical global gain and time-gain compensation settings.

**Figure 1.**
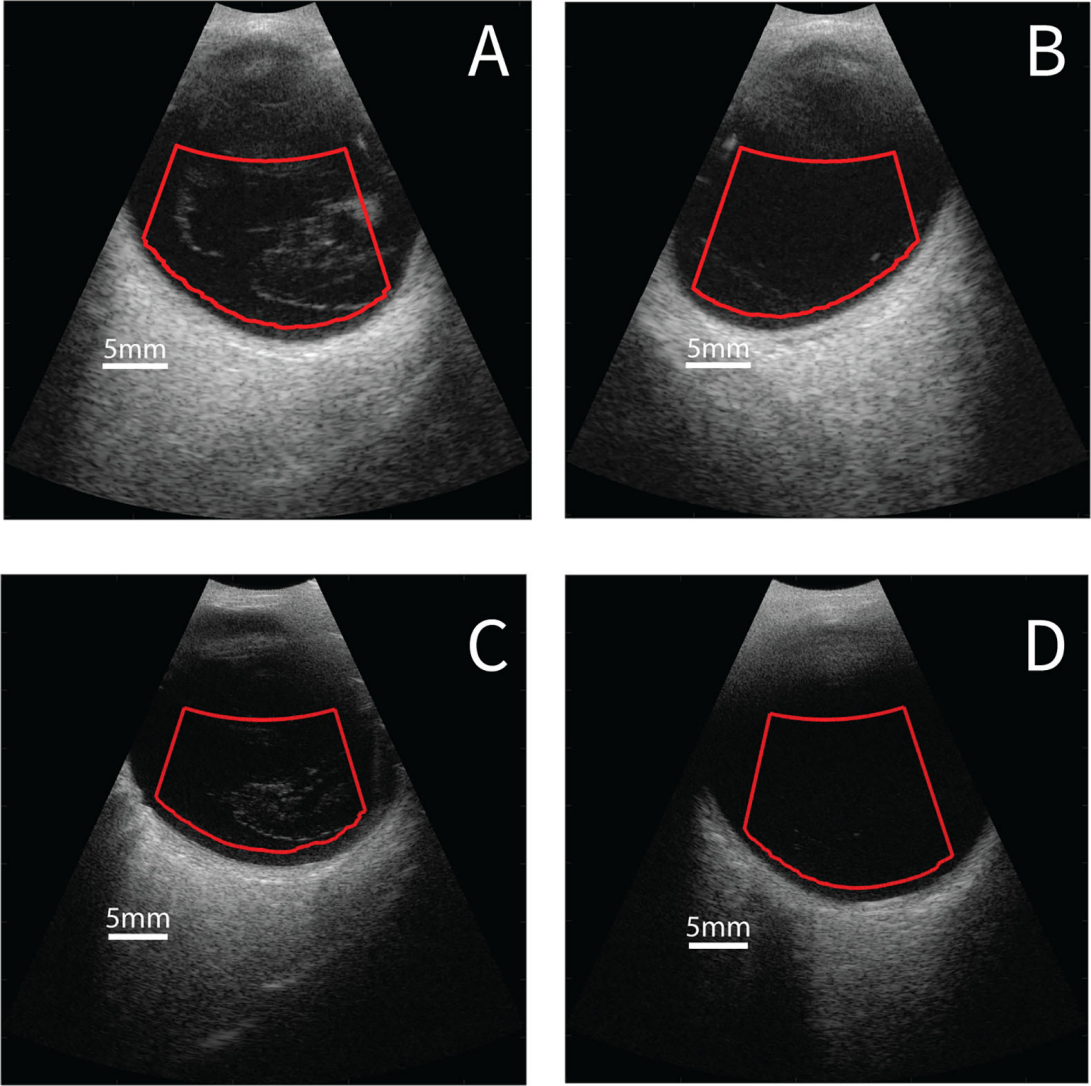
Representative B-mode images using the (**A,** **B**) 15-MHz transducer and (**C,** **D**) 20-MHz transducer. (**A,** **C**) Left eye of a patient with evidence of PVD and exhibiting many echodensities. (**B,** **D**) Right eye of a patient with no pathology and very few echodensities.

Each subject underwent ultrasonography after FrACT. Proparacaine 1% was used in both eyes to induce topical anesthesia. Systane gel (0.3% Hypromellose; Alcon, Inc., Fort Worth, TX, USA) was applied to the tip of the 15-MHz ultrasound probe. The probe was placed in direct contact with the nasal region of the globe to avoid acoustic attenuation through the eyelid. With the patient in temporal gaze, the contact point of the probe was posterior to the limbus to obtain a horizontal longitudinal scan plane through the central and premacular vitreous. Previous studies demonstrated that QUS parameters computed from data acquired in this scan plane were significantly correlated with visual function.^6^^,^^7^^,^^10^^,^^11^ After scanning both eyes of a patient with the 15-MHz probe, data were acquired in the same way using the 20-MHz probe and the second scanner. Representative B-mode images acquired from the same eyes with each machine are shown in Figure 1.

### Depth-Dependent Amplitude Measurements

**Figure 2.**
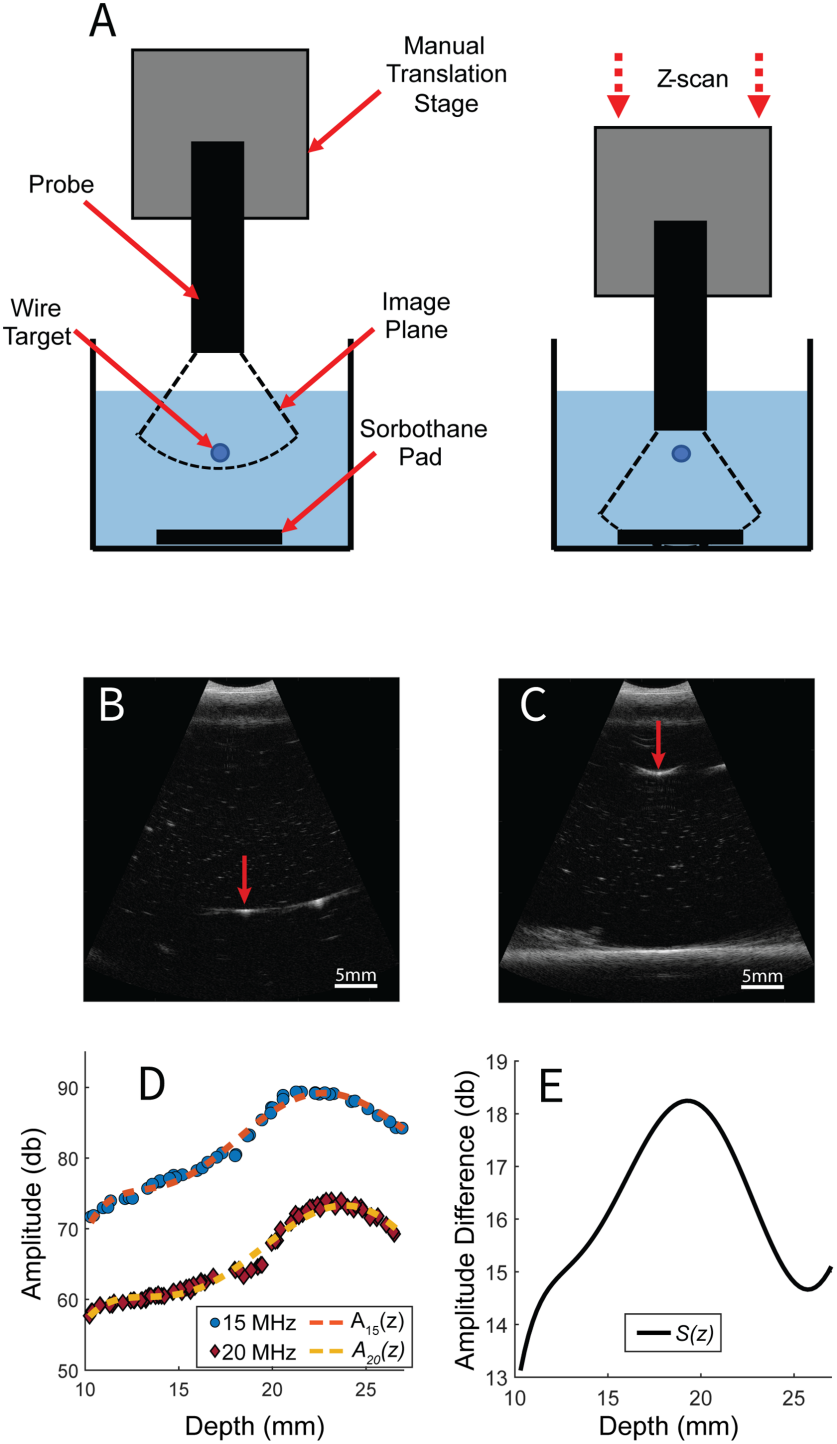
Z-scan measurements to determine depth-dependent scaling between the 15-MHz and 20-MHz transducers. (**A**) Illustration of the experimental apparatus. (**B,** **C**) *Red arrows* indicate the location of the suture target. At the beginning of the measurement, the target appears more distal (**B**) and gradually moves more proximal (**C**). (**D**) Maximum amplitude of the reflection at each depth for all three measurements with each transducer. Dashed lines are a sixth order polynomial fit. (**E**) Depth-dependent amplitude difference between the transducers.

As described above, the two machines have different engineering characteristics that affect the appearance of the B-mode images. For example, the higher center frequency of the 20-MHz probe produced images with finer spatial resolution, but lower sensitivity, relative to the 15-MHz probe. As a consequence, non-normalized QUS values obtained with the 2 probes from the same eye were expected to differ. A normalization step is therefore required to effectively compare the QUS parameters between probes. We hypothesized that a simple depth-dependent amplitude scaling term would correct for differences in transducer and machine characteristics and produce equivalent QUS parameter estimates. Measurements of the depth-dependent transducer sensitivity were made using the experimental apparatus illustrated in Figure 2a. For each measurement, one of the probes was attached to a manually controlled vertical translation stage. A 70-µm diameter wire target (ChromicGut suture; Ethicon Inc., Raritan, NJ, USA) was suspended in a container of degassed, deionized water. This type of suture was chosen as the wire target because of its small diameter and low reflectivity which avoided saturation of the recorded echo signal. An absorptive backing layer (Sorbothane Inc., Kent, OH, USA) was placed beneath the wire target to reduce reflections from the bottom of the container. Then, the manual stage was adjusted to orient the attached probe such that the wire target passed orthogonally through the image plane and initially visible in the far field of the B-mode. Data were acquired while the stage manually translated the ultrasound probe in a downward trajectory (z-scan) at a rate that ensured the wire target traversed the full axial range of the image plane within the 100-frame buffer limit. Z-scans were repeated three times for each machine. B-mode pixel values were converted to dB scale before the images were manually reviewed, segmented to identify the wire target in each frame, and the maximum reflection amplitude recorded at the wire depth. Figures 2b, c show representative B-mode images captured with the 20-MHz probe with the wire target visible in the distal and proximal axial range, respectively. For each probe, the depth-dependent amplitude measurements from the three z-scans were combined and fit to a sixth order polynomial to generate a smooth curve. The curves generated for the 15-MHz and 20-MHz probes are identified as *A*_15_(*z*) and *A*_20_(*z*), respectively. Finally, the depth-dependent scaling curve *S*(*z*) was computed as *S*(*z*) = *A*_15_(*z*) − *A*_20_(*z*) because the data were in the log-domain.

### Quantitative Ultrasound Processing

All data were acquired at VMR and transferred to Weill Cornell Medicine for offline QUS processing with automatic algorithms developed in MATLAB (2022b, The Mathworks, Natick, MA, USA). Datasets acquired with the 15-MHz probe contained up to 100 B-mode images captured at 16 frames/second whereas datasets acquired with the 20-MHz probe contained up to 200 frames captured at 16 frames/second. All images were manually reviewed for bad frames (e.g. reverberation artifacts or acoustically uncoupled) and frames containing artifacts were discarded. QUS processing was performed on the remaining frames in the same manner as previously described.^10^

A region of interest (ROI) was automatically generated within each artifact-free frame that encompassed the portion of the vitreous body determined to be within the depth-of-field of the ultrasound probe, as previously described.^10^ In each frame, the retinal surface was located using an edge detection algorithm and A-lines on the lateral edges of each B-mode were removed. The posterior edge of the ROI was defined to be approximately 1-mm anterior to the retinal surface (see Fig. 1). The proximal edge of the ROI was defined to start at 11.7 mm from the transducer because the image was not in focus anterior to that distance. Prior to processing each ROI, the pixel values were converted from digitized envelope values (0-255) to equivalent dB scale. The B-mode frames acquired with the 15-MHz probe spanned a dynamic range of 90 dB whereas the dynamic range of the 20-MHz probe spanned 80 dB. The pixel values were converted to dB scale by first dividing the pixel values by 255 and then multiplying by 90 dB (15-MHz data) or 80 dB (20-MHz data), which ensured consistent comparison of QUS results between the two machines.

B-mode images were processed using previously described QUS methods.^10^ After converting the pixel values to dB, each ROI was processed to compute three QUS parameters: mean (*M*), energy (*E*), and percentage of the ROI filled with echodensities (*P50*). Mean was defined as the sum of the pixel values within the ROI divided by the ROI area. Similarly, energy was defined as the sum of the squared pixel values divided by the ROI area. *P50* was calculated as the percentage of the ROI filled by clusters of echodensities filling at least 50 contiguous pixels and with magnitude ≥18 dB. This size was originally determined by inspecting the images and noting that echogenic regions smaller than this size and magnitude were typically associated with noise and not true echodensities.

The 20-MHz B-modes were processed a second time after normalizing the data to the 15-MHz machine. Two pre-preprocessing steps were applied: (1) the amplitude of the 20-MHz B-mode frames were increased based on a depth-dependent scaling curve, *S*(*z*), and (2) the magnitude threshold for the pixel clusters in the *P50* calculation was adjusted to be ≥10 dB. Depth-dependent scaling of the 20-MHz frames was performed on the raw B-mode data (i.e. before scan conversion) by adding *S*(*z*) to each A-line over the 10 to 28 mm axial range, chosen because it fully covers the ROI range. Moreover, inspecting histograms of pixel values within the ROIs of 20-MHz B-mode images suggested pixels with a value <10 dB were predominantly noise. Therefore, only pixels with values ≥10 dB were scaled*.* The B-mode data were then processed in the same way as above to obtain QUS parameters.

A fourth QUS composite score, *C*, was computed for each B-mode frame based on *M*, *E*, and *P50*. This composite score was intended to summarize characteristics of the echodensities with a single parameter^11^ and was found to correlate with axial length and CS.^6^ The composite score is defined as:

(2) $$\begin{array}{c} C=\frac{E}{2}+\left(M\times \;10\right)+\left(P50\;\times \;100\right).\; \end{array}$$

QUS processing was applied to each B-mode image in a dataset collected from a patient's eye. A final set of the four QUS parameters (*E*, *M*, *P50*, and *C*) was computed for each eye by averaging the parameters over all frames. For brevity, we introduce the notation *Q*_15_, *Q*_20_, and $Q_{20}^{*}$ to indicate QUS parameters estimated from the 15-MHz, 20-MHz, and 20-MHz normalized data, respectively, where *Q* ∈ {*M*, *E*, *P*50,  *C*}.

### Perturbation Analysis

Because frames with artifacts were excluded from processing, the final datasets contained different numbers of echo frames, potentially affecting the final averaged QUS parameter estimates. We therefore performed a perturbation analysis to determine the minimum number of frames required to obtain stable QUS parameters. The artifact free B-mode images were processed to compute the three QUS parameters (*M*, *E*, and *P50*). Each perturbation, *p*, was completed by computing the means of the QUS parameters over *N_F_* randomly selected frames:

(3) $$\begin{array}{c} \mu_{Q}^{p}=\frac{1}{N_{F}}\sum_{i\in R_{s}}Q_{i},\; \end{array}$$

where the notation *i* ∈ *R_s_* indicates the randomly selected subset of frames and *Q* corresponds to one of the three QUS parameters as defined in the previous section. After each perturbation, the mean, $\mu_{Q}^{N_{P}}$, and standard deviation, $\sigma_{Q}^{N_{p}}$, were found for each QUS parameter and used to compute the coefficient of variation (CV) as $CV_{Q}^{N_{F}}=\mu_{Q}^{N_{p}}$/$\sigma_{Q}^{N_{p}}*100\%$. The mean and standard deviation of the CV was then calculated across all perturbations, *N_p_*. As the number of randomly selected frames increases, it is expected that the CV will decrease, and the mean will converge to the “true” mean. A total of 11 perturbation analyses were performed, with *N_F_* ∈ {1,  5,  10,  15,  20,  25,  30,  35,  40,  50,  60} and *N_p_* = 1000. The minimum number of frames was identified as the smallest value of *N_F_* where the CV of all 3 QUS parameters decreased to less than 5%.

### Reproducibility

Reproducibility of the QUS measurements was tested by scanning seven eyes from four additional subjects (2 aged 33-34 and 2 subjects aged 70-72). Each eye was scanned three times sequentially. The mean, standard error of the mean (SEM), and CV were computed for all QUS parameters in each dataset. Intraclass correlation was used to determine the reproducibility of each QUS parameter. Reproducibility was reported as 100% CV (%) and deemed satisfactory if the intraclass correlation (ICC) exceeded 0.80.

### Statistical Analyses

QUS parameters estimated from data captured with the two probes were compared using linear regression and Pearson correlation (*Q*_15_ vs *Q*_20_, and *Q*_15_ vs. $Q_{20}^{*}$). Linear correlations were also used to compare CS to *Q*_15_, *Q*_20_, and $Q_{20}^{*}$. Each eye was treated as independent. Pearson correlation coefficients computed between CS and *Q*_15_, *Q*_20_, or $Q_{20}^{*}$ were compared using Steiger's two-sided z-test.^22^ This test was used to determine if QUS parameters from the two probes were equally correlated with CS. The level of significance was set to 0.05 for all statistical tests. Statistical analyses were performed using Python 3.9.7 with SciPy 1.7.1.

## Results

### Depth-Dependent Amplitude Normalization

Results of the depth-dependent amplitude measurements with both ultrasound probes are shown in Figure 2d. The sixth order polynomials *A*_15_(*z*) and *A*_20_(*z*) provide excellent fits to the measurements indicated by the scatterplot markers. Furthermore, the measurements exhibit very little dispersion around the fit curve, implying the separate z-scans were consistent measurements with small variance. Figure 2c contains a plot of *S*(*z*), the depth-dependent amplitude scaling factor. The peak of *S*(*z*) at *z* ≈  20 mm corresponds to the focal length of the 15-MHz probe. Similarly, the local minimum located at *z* ≈ 25 mm is near the focal length of the 20-MHz probe.

### Minimum QUS Frames From Perturbation Analysis

**Figure 3.**
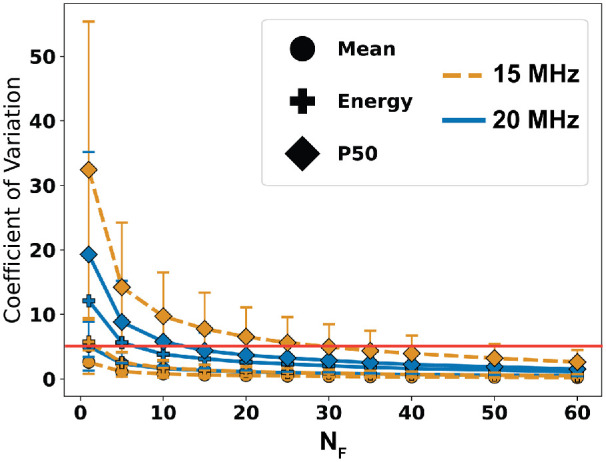
Coefficient of variation computed for the three QUS parameters plotted against the number of randomly selected frames during the perturbation analysis. Error bars represent the standard deviation of the CV and the red horizontal line indicates a CV of 5%. All parameters achieve a CV < 5% when 30 random frames are selected.

The CV values of *M*, *E*, and *P50* found through perturbation analysis are plotted against the number of randomly selected frames *N_F_* in Figure 3. All CV values decrease to below 5% when *N_F_* = 30. Therefore, at least 30 artifact-free frames are necessary to obtain consistent QUS parameter estimates. (The composite score *C* was omitted from the plot because it is derived from the other three parameters although the associated CV was also less than 5% at *N_F_* = 30.)

### Correlation of QUS With CS

Based on the criterion of at least 30 artifact-free frames for consistent QUS results, 10 eyes were omitted from further QUS processing. A total of 37 eyes from 22 patients were used in the final statistical analyses pertaining to QUS parameters.

**Figure 4.**
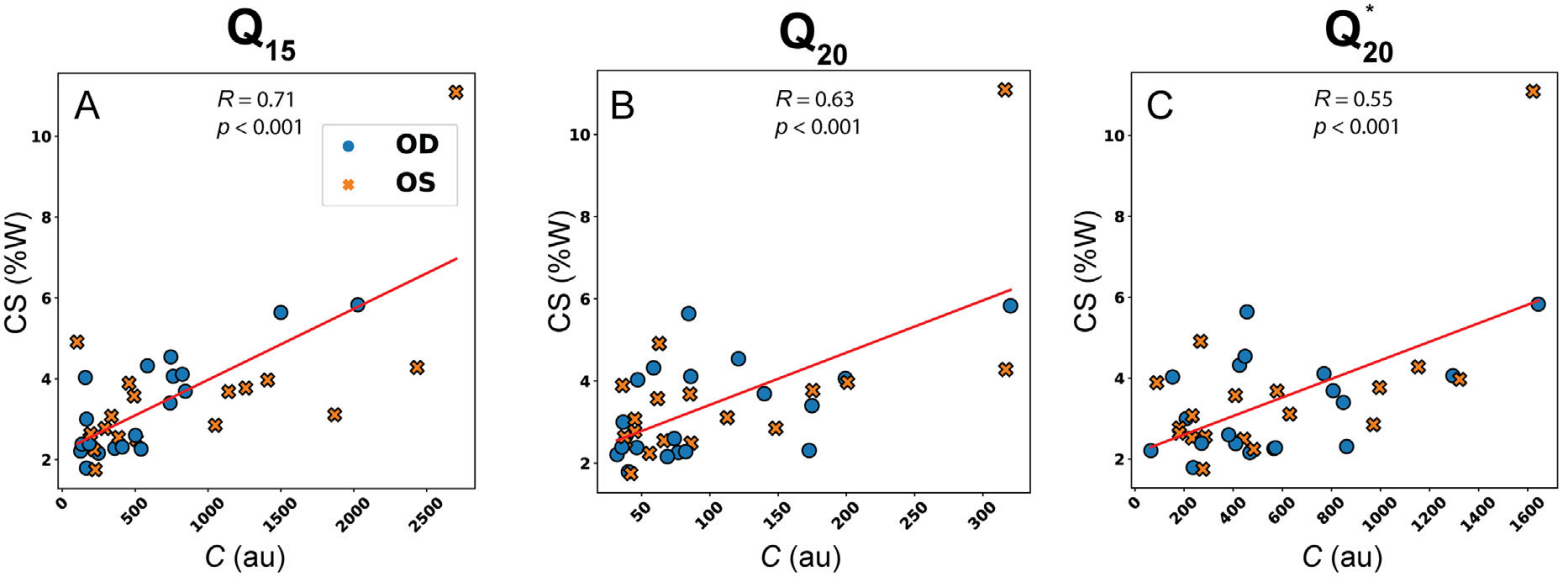
Representative scatterplots and correlations of CS with *C*. Markers specify right (OD) and left (OS) eyes for illustrative purposes only. *C* summarizes the three QUS parameters computed from (**A**) 15-MHz, (**B**) 20-MHz, and (**C**) normalized 20-MHz echo data.

Figure 4 displays representative plots of CS against *C* for *Q*_15_, *Q*_20_, and $Q_{20}^{*}$. The red line indicates the best linear fit. All Pearson correlation values in Table 1 indicate the statistically significant correlation between all QUS parameters and CS. The strongest correlations appear for *P50* (*R* = 0.72,  *p* < 0.001) and *C* (*R* = 0.71,  *p* < 0.001) computed from 15-MHz scan data. Conversely, the weakest correlations were found when comparing CS to *P50* (*R* = 0.57,  *p* < 0.001) and *C* (*R* = 0.55,  *p* < 0.001) computed from normalized 20-MHz echo data.

**Table 1.** Pearson Correlation Coefficients and *P* Values Computed From Linear Regression Analysis Comparing QUS Parameters to CS

	*Q*_15_		*Q*_20_		$Q_{20}^{*}$	
	*R*	*P* Value	*R*	*P* Value	*R*	*P* Value
*M*	0.62	<0.001	0.62	<0.001	0.64	<0.001
*E*	0.66	<0.001	0.63	<0.001	0.66	<0.001
*P50*	0.72	<0.001	0.62	<0.001	0.57	<0.001
*C*	0.71	<0.001	0.63	<0.001	0.55	<0.001

### Correlation of QUS Between Clinical Machines

Table 2 contains the linear correlation and *P* values among the three QUS parameters and composite score computed from B-mode data acquired with the two probes. Comparisons were made with and without normalization applied to the 20-MHz data. All correlations were statistically significant and achieved *R* ≥ 0.79.

**Table 2.** Pearson Correlation Coefficients and *P* Values Computed From Linear Regression Analyses Comparing QUS Parameters Computed From 15-MHz and 20-MHa Scan Data

	*Q*_15_ vs. *Q*_20_		Q15 vs .Q20*	
	*R*	*P* Value	*R*	*P* Value
*M*	0.82	<0.001	0.82	<0.001
*E*	0.83	<0.001	0.83	<0.001
*P50*	0.85	<0.001	0.83	<0.001
*C*	0.85	<0.001	0.79	<0.001

**Figure 5.**
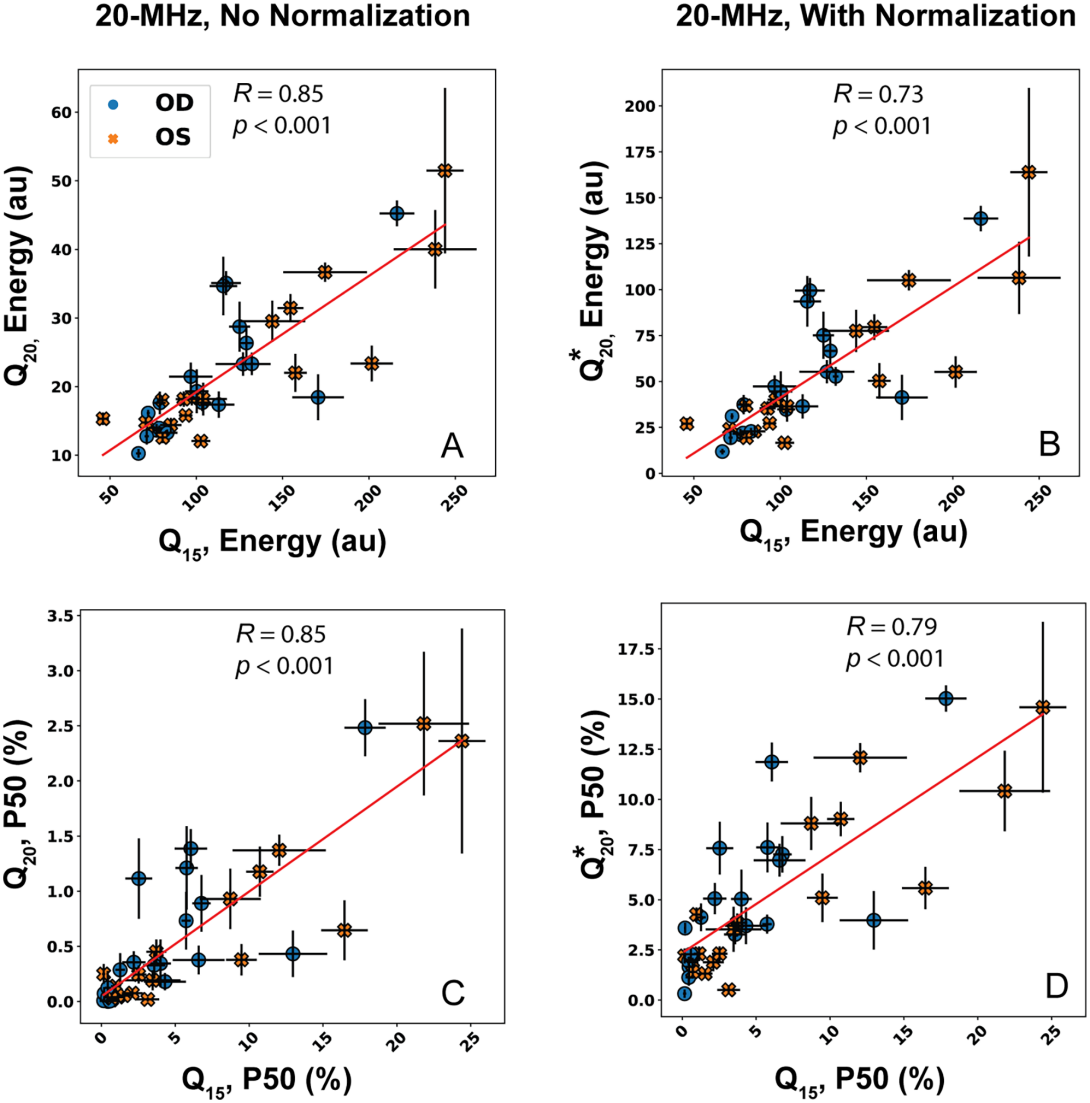
Correlations between the QUS parameters (**A,** **B**) *E* and (**C,** **D**) *P50* estimated from B-mode data acquired with the 15-MHz and 20-MHz probes. QUS parameters computed from 15-MHz data always appear on the x-axis. (**A,** **C**) The 20-MHz data were processed in the same manner as the 15-MHz data. (**B,** **D**) The 20-MHz data were normalized prior to QUS processing.

Figure 5 shows representative scatterplots of *E* and *P50* comparing 20-MHz data against the 15-MHz data. The red line indicates the line of best fit. Variability of the points around the linear fit appeared to remain consistent for *E* and *M* (not shown) with or without normalization applied to the 20-MHz datasets. The variability increased for *P50* and *C* (not shown), although *C* is highly dependent on *P50* as defined in Equation 2. Interestingly, *R* slightly decreased for *P50* and *C* when normalization and *P50* correction were applied.

To determine if the difference in Pearson correlations between CS and *Q*_15_, *Q*_20_, or CS and $Q_{15},\;Q_{20}^{*}$ were significant, a Steiger z-test was performed using the dependent correlation between QUS parameters listed in Table 2. All *P* values from the Steiger z-tests are compiled in Table 3. Pearson correlations between CS and *Q*_15_ or *Q*_20_ were found to be statistically similar (*p* > 0.14 for all). When comparing Pearson correlations among CS, *Q*_15_, and $Q_{20}^{*}$, correlations for P50 were found to be significantly different (*p* = 0.04), but *M* (*p* = 0.71), *E* (*p* = 0.42), and *C* (*p* = 0.10) had similar correlations.

**Table 3.** Comparison of Pearson Correlation Values of CS to QUS Parameters Estimated From 15-MHz and 20-MHa Datasets

	*Q*_15_ vs. *Q*_20_	Q15 vs .Q20*
*M*	1.00	0.80
*E*	0.69	1.00
*P50*	0.14	0.04
*C*	0.23	0.10

Statistical significance indicates the Pearson correlations differ.

### Reproducibility

**Figure 6.**
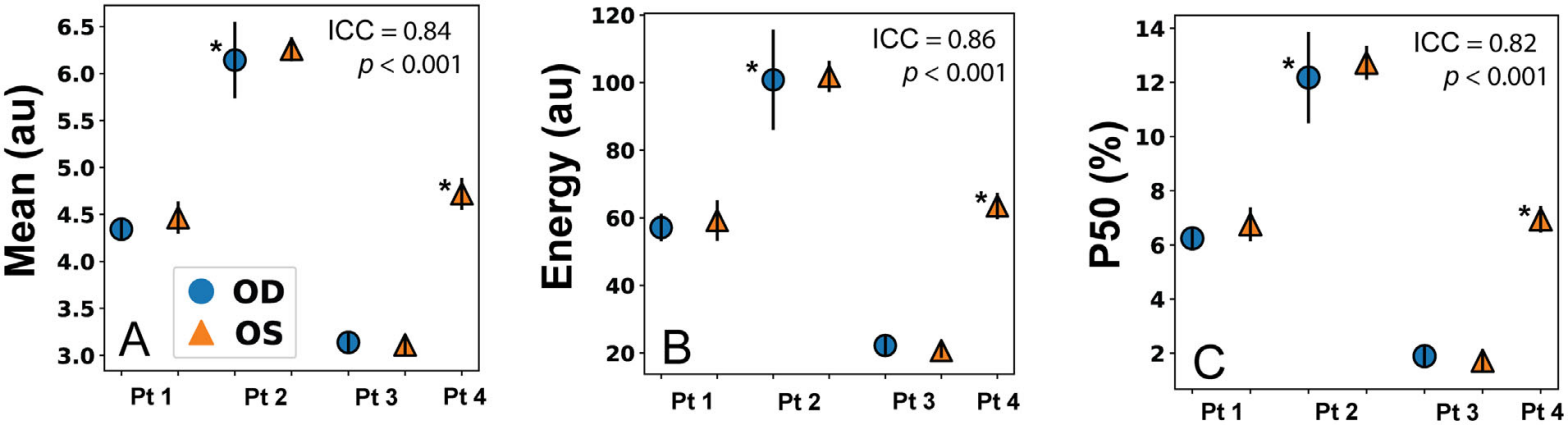
Results of reproducibility study with no normalization applied to 20-MHz B-mode data. Points are the mean parameter values computed from three measurements on each eye. Error bars represent standard error of the mean. Eyes with an asterisk (“*”) symbol next to the marker indicate presence of PVD.

Figure 6 contains plots of the mean and SEM for the three QUS parameters computed from repeated measurements of seven eyes. The variance of all measurements was reasonably small except for the right eye of one patient diagnosed with posterior vitreous detachment. Nevertheless, the reproducibility values and ICCs shown in Table 4 demonstrate satisfactory reproducibility (ICC > 0.80) for all 4 parameters when the 20-MHz probe was used. Reproducibility for the 15-MHz probe was shown in previous studies.^7^^,^^10^^,^^21^ Reproducibility was significant with and without normalizing the B-mode data.

**Table 4.** Reproducibility of the QUS Parameters Was Evaluate by Rep. (100% CV) and Intraclass Correlation

	No Normalization				With Normalization			
	Rep.	ICC	*P* Value	98% CI	Rep.	ICC	*P* Value	95% CI
*M*	96.3	0.84	<0.001	0.84–1.0	93.9	0.84	<0.001	0.83–1.0
*E*	89.7	0.86	<0.001	0.75–1.0	83.8	0.86	<0.001	0.78–1.0
*P50*	52.3	0.82	<0.001	0.25–0.99	78.0	0.89	<0.001	0.83–1.0
*C*	79.3	0.82	<0.001	0.38–0.99	80.0	0.89	<0.001	0.83–1.0

Three scans were performed on 7 eyes from a cohort of 4 subjects. Both Rep. and ICC were evaluated with and without normalizing the 20-MHz B-mode data.

CI, confidence interval.

## Discussion

QUS provides an objective quantification of vitreous echodensities that correlate with visual function, as measured by CS, and QOL, as assessed with the NEI VFQ. CS measures, VFQ scores, and QUS parameters not only provide quantitative metrics for determining when a patient would benefit from treatment, but also outcome measures of therapy. Previous studies established the correlation between light scatting and QUS,^9^ as well as between QUS parameters and CS or VFQ using image data acquired with a single ultrasound probe and clinical ophthalmic machine.^6^^,^^10^^–^^12^ Results of the present study demonstrate that QUS parameters derived from images acquired with two different machines and imaging probes are highly correlated and perform equally well.

Although QUS parameters were highly correlated between machines (*R* ≥ 0.82 in all cases), there was a difference in magnitude. This was expected given the fundamentally different properties of the single-element transducer in the 15-MHz probe and the 5-ring annular array in the 20-MHz probe. Attempting to account for system effects through the depth-dependent scaling factor and *P50* correction brought the magnitudes of QUS parameters nearer, but they still differed significantly. We hypothesize that adjusting the amplitude of the 20-MHz data corrected for differences in transducer sensitivity, but not for acoustic beam properties. For example, echodensities captured with the 15-MHz transducer appear larger compared to the same echodensities scanned with the 20-MHz probe. Given that *M*, *E*, and *P50* are influenced by the apparent size of echodensities relative to the ROI area, the fundamental differences in beam properties must be considered to properly normalize QUS parameters across machines. Alternatively, results of the linear regression analysis could be used to define a first order transformation of the QUS parameters, although this method ignores the physics of the problem. Nevertheless, QUS parameters from both machines achieved statistically significant correlation with CS. Moreover, all Pearson correlation values between CS and *Q*_15_ or CS and *Q*_20_ were statistically similar, implying there is no difference in the efficacy of QUS parameters to characterize the effect of echodensities on vision based on machine or probe. However, if the parameters are used to determine when clinical treatment is appropriate, the thresholds must be adjusted based on the imaging probe and machine.

Normalizing the 20-MHz data had some interesting effects on the correlations between QUS parameters and CS. Specifically, the correlation of *P50* (and *C* given its strong dependence on *P50*) with CS decreased slightly after normalization. Similarly, the correlation between *P50* and *C* in *Q*_15_ and $Q_{20}^{*}$ marginally decreased with normalization. Given that normalization was depth-dependent and the computation of *P50* is highly nonlinear, it is difficult to identify precisely why data normalization affected the correlations in this way. Accounting for differences in the acoustic beam properties during normalization may have produced a marginal increase in correlation with CS but results of this study do not suggest the additional effort is necessary: QUS parameters computed from raw 20-MHz scan data are just as effective at characterizing vitreous echodensities as QUS parameters from 15-MHz or 20-MHz data.

Variability in QUS parameters arises primarily from the inability to capture the same scan plane across all frames. Patient eye movement and differences in placement of the probe upon the globe contribute to disparities in the orientation of the scan plane through the eye. The effect of patient eye movement on QUS parameters is most apparent by the error bars in Figure 5. It is not possible to completely eliminate eye motion and therefore a criterion for data collection is necessary to obtain consistent QUS parameters. Results of the perturbation analysis suggest that collecting at minimum 30 image frames provides sufficient data to compute consistent QUS parameter values. This data collection standard will be used for guiding future studies and clinical evaluations. A minimum frame requirement reduces the potential effects of microsaccades, but large eye movements should still be avoided to minimize QUS parameter variance. Typically, up to 100 frames can be captured with the AVISO machine, whereas up to 200 frames can be captured with the ABSOLU machine.

The QUS vitreous echodensity parameters from both ophthalmic ultrasound machines correlated with visual function. Differences in parameter magnitudes between the two machines arose because we were unable to apply backscatter-based normalization to the log-compressed image data. The major limitation of the current study and QUS methods is the inability to obtain RF echo data collected with the 15-MHz probe. In other organs and tissue types, QUS parameters computed from RF data and the backscatter coefficient account for machine properties by using measurements of a planar reflector or calibration phantom.^13^^,^^17^ Normalizing the data in this way allows for estimating “true” properties related to tissue microstructure by removing system effects from the measured data. Our goal is to create novel QUS methods for evaluating VDM based on backscattering models that exploit the information encoded in raw RF echo data that are more robust, user- and machine-independent. Modern clinical scanners, such as the Absolu, can provide access to the RF echo data necessary to develop these techniques.

In summary, QUS parameters computed from log-compressed envelope data were equally effective at assessing vitreous echodensities at 15 MHz and 20 MHz using 2 different clinical ophthalmic ultrasound machines. Differences in the parameter magnitudes across machines could not be eliminated by depth-dependent scaling of the envelope data. QUS methods based on advanced scattering models and operating on the RF data may provide true machine- and operator-independent methods to objectively evaluate vision degrading myodesopsia using QUS.
